# The role of gut microbes in drought adaptation in the five-toed jerboa (*Orientallactaga sibirica*)

**DOI:** 10.1186/s12866-025-04204-z

**Published:** 2025-08-05

**Authors:** Haoting Zhang, Dongyang Chu, Haiwen Yan, Shanshan Sun, Xiaodong Wu, Heping Fu, Shuai Yuan

**Affiliations:** 1 College of Grassland Science, Inner Mongolia Agricultural University, Hohhot, 010011 China; 2 Key Laboratory of Grassland Rodent Ecology, Rodent Pest Control at Universities of Inner Mongolia Autonomous, Hohhot, 010011 China; 3 Key Laboratory of Grassland Resources, Ministry of Education P.R. of China, Hohhot, 010011 China

**Keywords:** Five-toed Jerboa, *Orientallactaga sibirica*, Drought adaptation, Gut microbiota, Water stress

## Abstract

The composition of the gut microbiota of animals is closely linked to host health and environmental adaptability. It can increase the phenotypic plasticity of animals, thereby improving their ability to thrive in harsh environments. The five-toed jerboa (*Orientallactaga sibirica*) is widely distributed in various regions, particularly in western Inner Mongolia and Xinjiang, which are located in the arid western part of China, and is capable of tolerating an arid environment. However, the relationships between the five-toed jerboa’s adaptation to such conditions and its gut microbiota have not yet been studied. In this study, we aimed to investigate the relationships between the gut microbiota of the five-toed jerboa and their adaptations to drought and water scarcity. Five-toed jerboa individuals were subjected to two treatments: water deprivation (experimental group, EG) and normal drinking water intake (control group, CG). Fecal samples from both groups were collected for sequencing and analysis of the V3-V4 variable region using 16 S rRNA. The results indicated that while the microbial composition is similar, there are differences in the relative abundance of microbes. The dominant bacteria in both groups were Firmicutes and Bacteroidota. The relative abundances of various genera within the bacterial community were significantly altered, with the abundance of *Lactobacillus* being most affected. The alpha diversity of the gut microbiota significantly differed between the EG and CG on the 5th and 11th days of water stress, with the EG showing significantly greater diversity than the CG (ACE, Chao and Shannon index) (*P* < 0.05). PICRUSt prediction revealed that the abundance of metabolism-related genes remained relatively consistent in the CG; however, in the EG, the abundance of genes associated with metabolism and cellular processes increased as the duration of stress increased. The five-toed jerboa adapted to water stress conditions by increasing the abundances of short-chain fatty acid-producing bacterial genera and altering the diversity of its gut microbiota. The results of this study suggest that five-toed jerboa may adapt to arid and water-scarce environments by increasing the abundance of beneficial bacteria in their gut microbiota.

**Supplementary Information:**

The online version contains supplementary material available at 10.1186/s12866-025-04204-z.

## Introduction

Animals undergo behavioral, morphological, and physiological changes to adapt to their environment through long-term natural selection [1–4]. A diverse array of microorganisms inhabit the animal intestinal tract, forming a complex and unique ecosystem that is intricately linked to the physiological functions of the host organism [5]. Gut microbes play crucial roles in the digestion of food [6–8], in nutrient absorption [9–11], and in metabolic immunity [12]. This role is influenced by the composition and structure of the microbial community. Additionally, gut microbes evolve synergistically with the host during adaptation to environmental changes [13, 14], establishing an interdependent relationship that significantly contributes to the host’s adaptability [5, 15, 16]. Consequently, the composition and structure of the gut microbial community can serve as indicators of the physical condition of a host and its ability to adapt to its living environment [13, 17]. Furthermore, studying changes in the community of gut microbes in animals can help answer questions about animal adaptation [18].

Animals in arid environments must cope with water scarcity [19], extreme temperatures [20], and food shortages [21, 22]. To adapt to these harsh conditions, animals living in arid regions have evolved various strategies, such as changing their fur color [2], entering a state of summer hibernation [23], and lowering their resting metabolic rates [24]. Research has shown that environmental conditions not only affect mammals but also influence their symbiotic microbiota [25–28]. Changes in precipitation in arid regions impact plant communities and productivity, alter host gut microbes and affect rodent adaptation [29]. Moisture is the most critical limiting factor in arid environments and is essential for animal survival [30–32]. A recent study revealed that different sources of water influence animal gut microbes, leading to variations in the composition of gut microbial communities on the basis of the amount of water consumed [33, 34]. While moisture plays a significant role in shaping animal gut microbiomes, relatively few studies have directly used water as a control variable to investigate the effects of water stress on these microbial communities. The potential coevolution of gut microbes in arid environments, along with the gut microbiota and host during drought adaptation, remains poorly understood.

The five-toed jerboa (*Orientallactaga sibirica*) is widely distributed across China and inhabits semihumid, semiarid, and arid zones. Its primary areas include the Loess Plateau and regions to the north, particularly the arid western parts of Inner Mongolia and Xinjiang. This species exhibits remarkable adaptability, thriving in various habitats, such as grasslands, deserts, and the Gobi [35]. Notably, its coat color and body size vary according to its habitat, enabling it to survive under the harsh conditions of arid climates [36, 37]. In addition to metabolism, phenotype, and the genome, a crucial factor in the adaptation of the five-toed jerboa to arid environments is their gut microbiome. Research has shown that microbiome interactions can regulate the health of organisms and facilitate adaptation to environmental changes [17]. Some studies on the five-toed jerboa have been conducted, but the research on their gut microbiome remains relatively limited. Existing studies have primarily concentrated on the effects of diet on the composition and diversity of gut microbes in jerboas [38, 39]. Therefore, this study aims to investigate how the gut microbes of five-toed jerboa change under conditions of water scarcity and the role they play in host adaptation to arid environments. We conducted a water stress experiment involving the five-toed jerboa, establishing a water-fasting stress experimental group and a control group receiving a normal amount of drinking water. Fecal samples were collected from both groups for 16 S rRNA sequencing. We hypothesized that differences in the composition, abundance, and diversity of gut microorganisms between the water-stressed and control groups would increase the ability of the host to adapt to drought conditions.

## Materials and methods

### Study site, animals and experimental design

Twelve healthy adult five-toed jerboa (average weight = 101.8 ± 7.8 g) were captured via the cage-trapping method in the study area in May 2021. The study area is located in a typical desert region on the eastern edge of the Tengger Desert in Inner Mongolia, China (E104°10′–105°30′, N37°24′–38°25′). This area experiences a continental climate characterized by cold, dry winters and warm summers. The annual precipitation ranges from 45 mm to 215 mm, with approximately 70% of this precipitation occurring between June and September. Potential evaporation rates vary from 3,000 to 4,700 mm, and the annual frost-free period lasts 156 days. The animals were housed in a simulated natural environment at the Desert Ecology and Rodent Pest Control Research Base of Inner Mongolia Agricultural University. They were provided with standard mouse food as their primary diet under natural light conditions and had free access to water. The jerboas were fed for one week to help them acclimate and recover from the stress of capture, followed by an additional week to stabilize. During this period, food intake was measured every two days, and body weight was recorded daily until stabilization was achieved.

After weight stabilization, a water control experiment was conducted, in which the subjects were divided into two experimental groups: (1) the experimental group (EG), which was prohibited from drinking water, and (2) the control group (CG), which had normal access to water. In June and July, twelve five-toed jerboas with stable body weights were subjected to water stress over a total period of 11 days. The experimental group was fed standard mouse food without water, whereas the control group received the same food along with a normal supply of water. The standard diet did not contain moisture, meaning that the animals could not obtain water from their food. Body weight was measured daily (± 0.1 g) throughout this period. Fecal samples from the five-toed jerboa individuals were collected on the 1st, 5th, and 11th days. The feces from the EG were labeled EG1, EG5, and EG11, whereas those from the CG were labeled CG1, CG5, and CG11, according to the sampling periods. All fecal samples were collected under aseptic conditions and placed in 2 ml sterile freezing tubes. The samples were then frozen in liquid nitrogen and stored at -80 °C. The collected fecal samples were sent to Shanghai Meiji Biopharmaceutical Science and Technology Co. for analysis of intestinal microbial amplicons. We euthanized five-toed jerboa using inhalation overdose of isoflurane. 3.5% isoflurane was used for induction. Euthanasia of five-toed jerboa by cervical dislocation after confirmation of unconsciousness. For small rodents weighing less than 200 g, cervical dislocation was performed by experienced personnel. This method was chosen due to its efficiency and minimal distress to the animal. Our study was approved by the Ethics Committee of Inner Mongolia Agricultural University under the approval number NND2017012.

### DNA extraction and PCR amplification

Total microbial genomic DNA was extracted from 36 samples using the E.Z.N.A.^®^ soil DNA Kit (Omega Biotek, Norcross, GA, U.S.) according to the manufacturer’s instructions. The quality and concentration of the DNA were determined by 1.0% agarose gel electrophoresis and a NanoDrop^®^ ND-2000 spectrophotometer (Thermo Scientific Inc., USA), and the samples were stored at -80 °C until further use. The hypervariable region V3-V4 of the bacterial 16 S rRNA gene was amplified with the primer pairs 338 F (5’-ACTCCTACGGGAGGCAGCAG-3’) and 806R (5’-GGACTACHVGGGTWTCTA AT-3’) [40] by an ABI GeneAmp^®^ 9700 PCR thermocycler (ABI, CA, USA). The PCR mixture included 4 µL of 5 × Fast Pfu buffer, 2 µL of 2.5 mM dNTPs, 0.8 µL of each primer (5 µM), 0.4 µL of Fast Pfu polymerase, 10 ng of template DNA, and ddH2O to a final volume of 20 µL. The PCR amplification cycling conditions were as follows: initial denaturation at 95 °C for 3 min; 27 cycles of denaturation at 95 °C for 30 s, annealing at 55 °C for 30 s, and extension at 72 °C for 45 s; and a single extension at 72 °C for 10 min, ending at 4 °C. All samples were amplified in triplicate. The PCR product was extracted from a 2% agarose gel and purified using the AxyPrep DNA Gel Extraction Kit (Axygen Biosciences, Union City, CA, USA) following the manufacturer’s instructions and quantified via a Quantus™ Fluorometer (Promega, USA).

Purified amplicons were pooled in equimolar amounts and paired-end sequenced on an Illumina MiSeq PE300 platform (Illumina, San Diego, USA) according to standard protocols by Majorbio Bio-Pharm Technology Co. Ltd. (Shanghai, China). The raw sequencing reads were deposited into the NCBI Sequence Read Archive (SRA) database (accession number: PRJNA1240187).

### Illumina miseq sequencing

Raw FASTQ files were demultiplexed using an in-house Perl script and then quality-filtered by fastp version 0.19.6 [41] and merged by FLASH version 1.2.7 [42] with the following criteria: (i) the 300 bp reads were truncated at any site receiving an average quality score of < 20 over a 50 bp sliding window, and truncated reads shorter than 50 bp were discarded; reads containing ambiguous characters were also discarded; (ii) only overlapping sequences longer than 10 bp were assembled according to their overlapped sequence. The maximum mismatch ratio of the overlap region was 0.2. Reads that could not be assembled were discarded. (iii) Samples were distinguished according to barcode and primers, and the sequence direction was adjusted to include exact barcode matching and 2 nucleotide mismatches in primer matching. The optimized sequences were subsequently clustered into operational taxonomic units (OTUs) using UPARSE 7.1 [43, 44] at a 97% sequence similarity level. The most abundant sequence for each OTU was selected as a representative sequence. To minimize the effects of sequencing depth on alpha and beta diversity, the number of 16 S rRNA gene sequences from each sample was rarefied to 23,792, which still yielded an average Good’s coverage of 99.09%. The taxonomy of each OTU representative sequence was analyzed by RDP Classifier version 2.2 [45] against the 16 S rRNA gene database (e.g., Silva v138) with a confidence threshold of 0.7. Metagenomic function was predicted via Phylogenetic Investigation of Communities by Reconstruction of Unobserved States (PICRUSt2) [46], which is based on representative OTU sequences.

### Statistical analysis

Alpha diversity indices (Ace, Chao 1, Shannon, and Simpson indices) were calculated using Mothur software [47] (http://www.mothur.org/wiki/Calculators). The Wilcoxon rank-sum test was employed to analyze differences in alpha diversity between groups. The similarity of microbial community structures among samples was examined using principal coordinate analysis (PCoA) based on the weighted UniFrac distance algorithm combined with the PERMANOVA nonparametric test to assess the significance of differences in microbial community structure among sample groups. Additionally, linear discriminant analysis (LEfSe) [48] was utilized to evaluate differences in microbial diversity among sample groups. Effect size [38] (http://huttenhower.sph.harvard.edu/LEfSe) (LDA > 4, *P* < 0.05) was used to identify bacterial taxa with significant differences in abundance from the phylum to the genus level among the different groups.

## Results

### Weight change in five-toed Jerboa

**Fig. 1:**
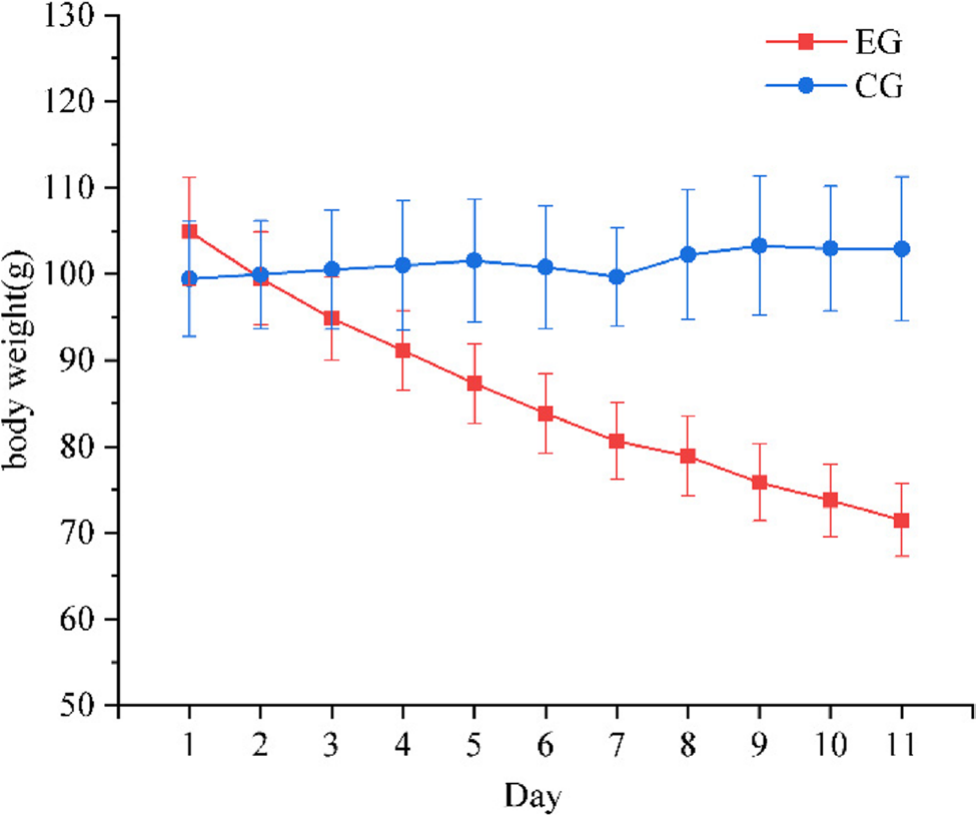
Changes in the body weights of five-toed jerboa in the water stress control and experimental groups (red: EG, blue: CG). CG stands for the normal drinking water control group, and EG stands for the water-stressed experimental group

Drought stress had a significant effect on the body weight of five-toed jerboa. Repeated-measures ANOVA was employed to assess the differences in the effects of EG and CG on the body weight of five-toed jerboa. An interaction between time and group was identified (*F*_day*group_ = 138.633, *P* < 0.001). A significant difference in body weights between the EG and CG groups was observed at various time points (*F* = 410.786, *P* = 0.038). Notably, a significant difference in body weights between the EG and CG groups emerged starting on day 4 (*F* = 6.717, *P* = 0.027) (Fig. 1).

### Sequencing results

The analysis of the 16 S rRNA sequencing results revealed a total of 1,371,390 sequences obtained from 36 samples, with an average of 38,094 sequences per sample and an average length of valid sequences measuring 420 bp. The rarefaction curve (Fig. S1) increased with sample size and reached a clear saturation point, indicating that the experiments provided sufficient sampling and sequencing depth to adequately represent the gut microbial community of the five-toed jerboa.

### Gut microbiome composition and OTUs between treatments

Valid sequences from all samples were clustered into OTUs with 97% similarity, resulting in a total of 781 OTUs classified into 13 phyla, 21 classes, 47 orders, 80 families, 158 genera, and 277 species. The top five phyla identified were Firmicutes, Bacteroidota, Actinobacteriota, Verrucomicrobiota, and Campylobacterota, while the top five genera included *Lactobacillus*, *norank_f__Muribaculaceae*, *Bacteroides*, *Bifidobacterium*, and *Lachnospiraceae_NK4A136_group*.

**Fig. 2:**
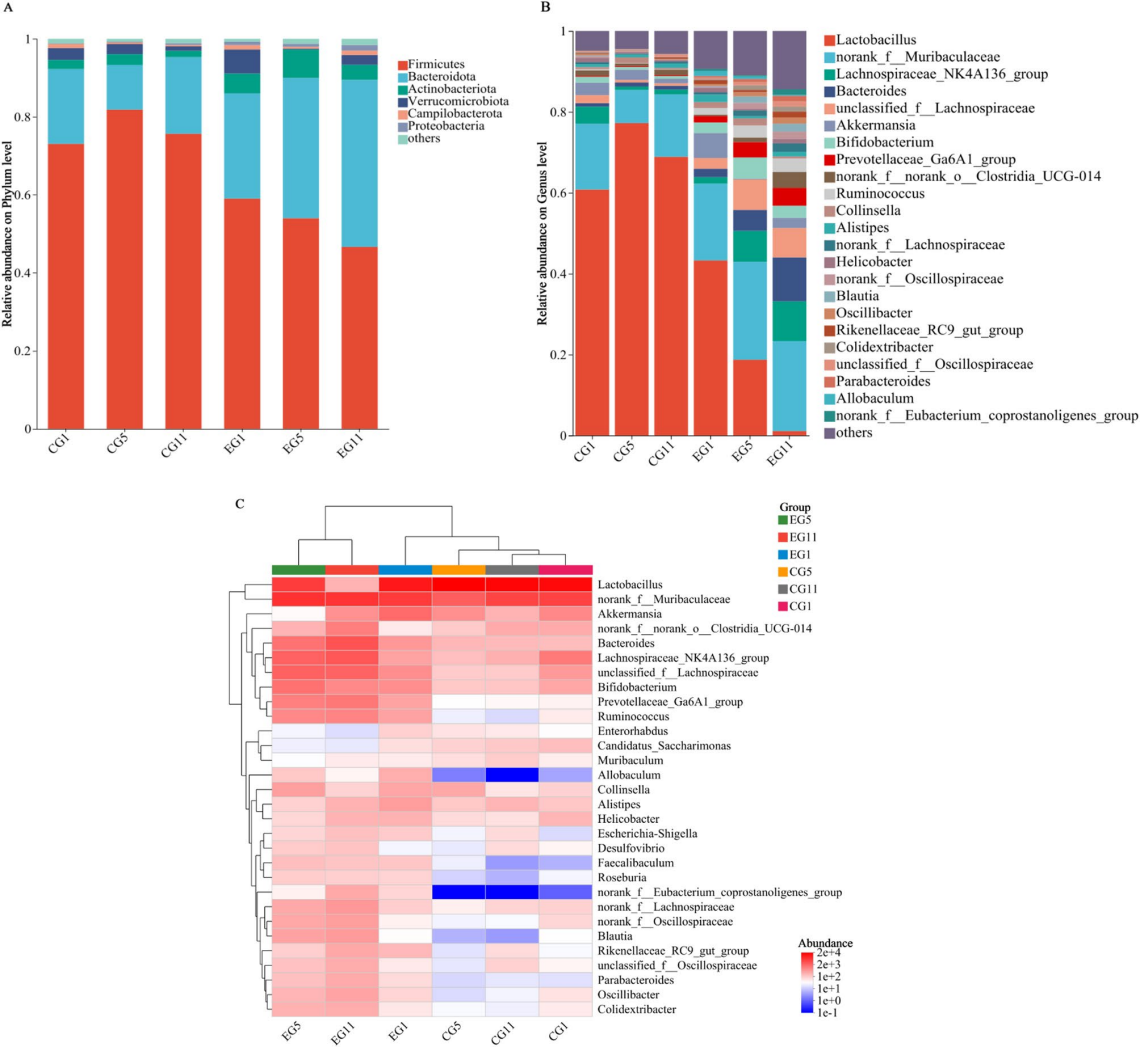
Gut microbiome composition of samples from different periods in the five-toed jerboa control group and the water stress group: (**A**) phylum level, (**B**) genus level. **C** Heatmap of the abundances of the top 30 genera according to sample type in the five-toed jerboa control group and the water stress group in different periods. CG represents the control group; EG represents the water stress group; CG1, CG5 and CG11 represent samples taken on Days 1, 5 and 11 in the control group; and EG1, EG5 and EG11 represent the samples taken on Days 1, 5 and 11 in the water stress group, respectively

At the phylum level, the gut microorganisms of the five-toed jerboa were predominantly Firmicutes and Bacteroidota, which together accounted for more than 85% of the community. This was followed by Actinobacteriota, Verrucomicrobia, Campilobacterota and Proteobacteria. A comparison of the community composition at the phylum level between the EG and CG revealed notable differences in proportions. The CG presented the highest relative abundance of Firmicutes, followed by Bacteroidota. Conversely, the relative abundances of Verrucomicrobia and Campilobacterota gradually decreased. The relative abundances of Firmicutes and Bacteroidota in the gut of the five-toed jerboa in the EG varied considerably, and the relative abundance of Firmicutes gradually decreased with increasing duration of water fasting, from 59.03 to 46.64%; the relative abundance of Bacteroidota gradually increased from 26.97 to 42.86%, and the relative abundance of Proteobacteria also increased gradually, with an increase in relative abundance of up to 1% (Fig. 2A).

At the genus level, the gut microbes of the five-toed jerboa primarily consisted of *Lactobacillus*, *norank_f_Muribaculaceae*, *Lachnospiraceae_NK4A136_group*, *Bacteroides*, *unclassified_f_Lachnospiraceae*, and others. In the CG, the dominant genera were *Lactobacillus*, *norank_f_Muribaculaceae*, and *Lachnospiraceae_NK4A136_group*, which collectively accounted for over 80% of the community, whereas the other genera were relatively less abundant. In contrast, the EG presented a greater relative abundance of various genera, with a notable decrease in *Lactobacillus* from 43.28 to 1.09%. Moreover, the relative abundances of *norank_f_Muribaculaceae*, *Lachnospiraceae_NK4A136_group*, *Bacteroides*, *unclassified_f_Lachnospiraceae*, and *Bifidobacterium* increased, whereas *Akkermansia* and *Collinsella* decreased in relative abundance (Fig. 2B). The top 30 genera in terms of abundance in the different treatment communities are displayed using community heatmap plots, in which the samples in the EG5 and EG11 groups clustered together. CG1, CG5, CG11, and EG1 clustered together, indicating that changes in the genera of the five-toed jerboa in the EG on Day 1 of water fasting were similar to those in the CG. The composition of the gut microbiota was similar in the EG on Days 5 and 11, and genus *Lactobacillus* had higher abundance in the drinking water group. The genera *Allobaculum*, *Faecalibaculum* and *norank_f__Eubacterium_coprostanoligenes_group* had opposite trends in terms of relative abundance changes in the CG and EG groups (Fig. 2C).

**Fig. 3:**
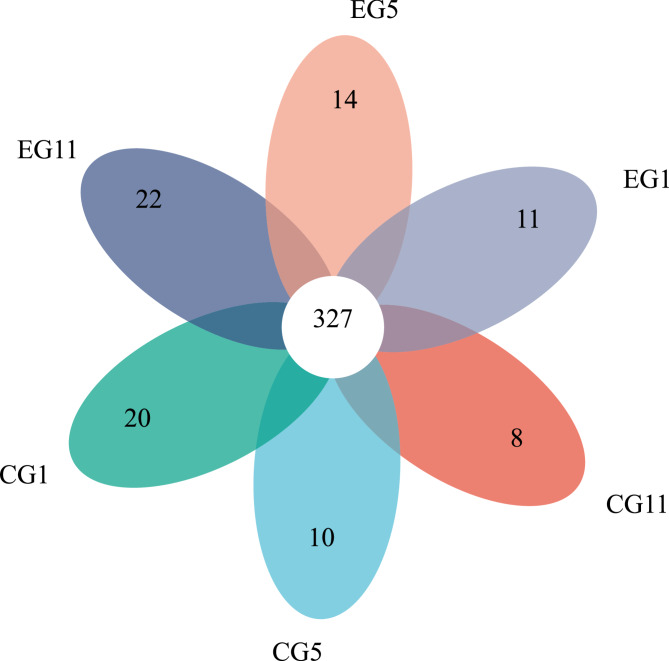
Venn diagram based on the OTUs of the EG and CG in different periods

A Venn diagram of the gut microbial community of the five-toed jerboa at the OTU level is shown in Fig. 6. The total number of OTUs identified in the gut microbes of the five-toed jerboa across different treatments and sampling times was 327. In the CG, the number of unique OTUs at various sampling times was 20, 10, and 8, indicating a decrease in unique OTUs over time. Conversely, in the EG, the number of unique OTUs at different sampling times was 11, 14, and 22, demonstrating an increase in unique OTUs as fasting time progressed (Fig. 3).

### Differences in gut microbial alpha and beta diversity between treatments

**Fig. 4:**
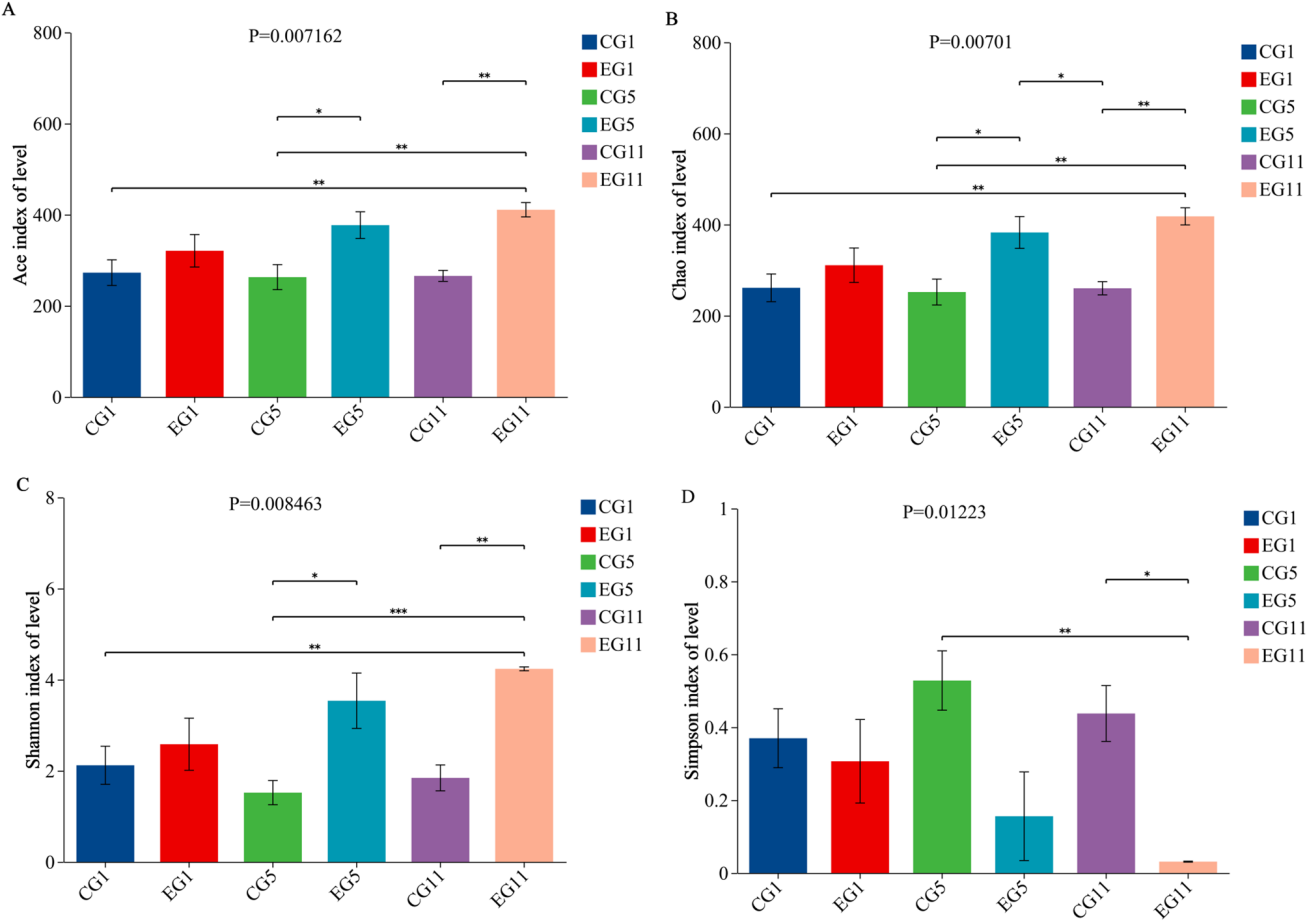
Alpha diversities of the gut microbiota differed between the EG and CG in different periods. **A** Ace. **B** Chao. **C** Shannon. **D** Simpson index

Analysis of the α diversity index revealed no significant differences among the various periods within each group, including the water fasting group and the control group. However, the α diversity of the gut microorganisms began to change in both the EG and CG on the fifth day of water fasting. Specifically, there were significant differences in the Ace, Chao, and Shannon indices between EG5 and CG5. On the eleventh day of water fasting, the α diversity indices (Ace, Chao, Shannon, and Simpson) were significantly different between EG11 and CG11, with the diversity of the gut microbiome being greater in the EG than in the CG (Fig. 4).

**Fig. 5:**
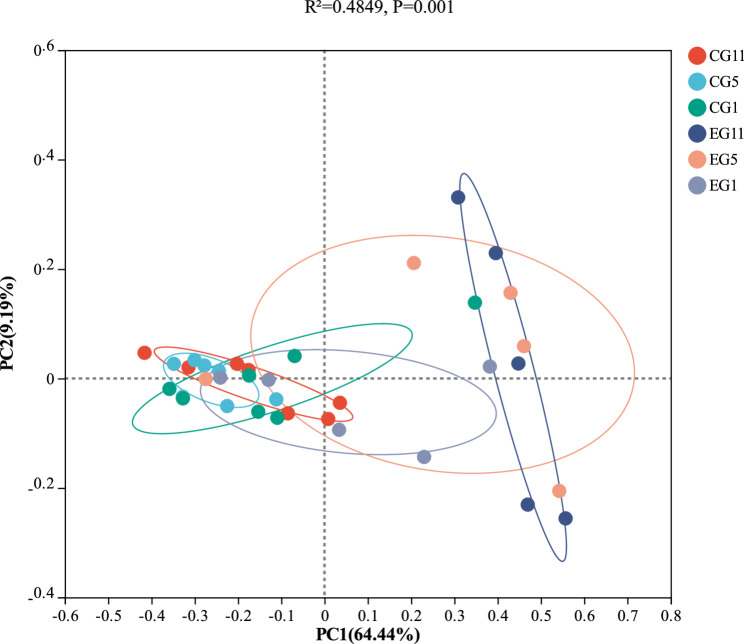
Analysis of the β diversity of the gut microbiota of five-toed jerboa at different sampling periods under different treatments

β diversity analysis was performed using principal coordinate analysis (PCoA) based on the Bray‒Curtis distance (Fig. 5), in which the horizontal coordinate PC1 accounted for 64.44% of the variation and the vertical coordinate PC2 explained 9.19% of the variation, with the two axes explaining 73.63% of the total variation (R² = 0.4849, *P* = 0.001). This analysis revealed that the gut microbial communities in the CG were more similar to one another in different periods than those in the EG were. Additionally, the gut microbial communities of EG5 and EG11 were closely related. PERMANOVA revealed that different treatments significantly influenced the gut microbial composition of five-toed jerboa (*P* = 0.001).

**Fig. 6:**
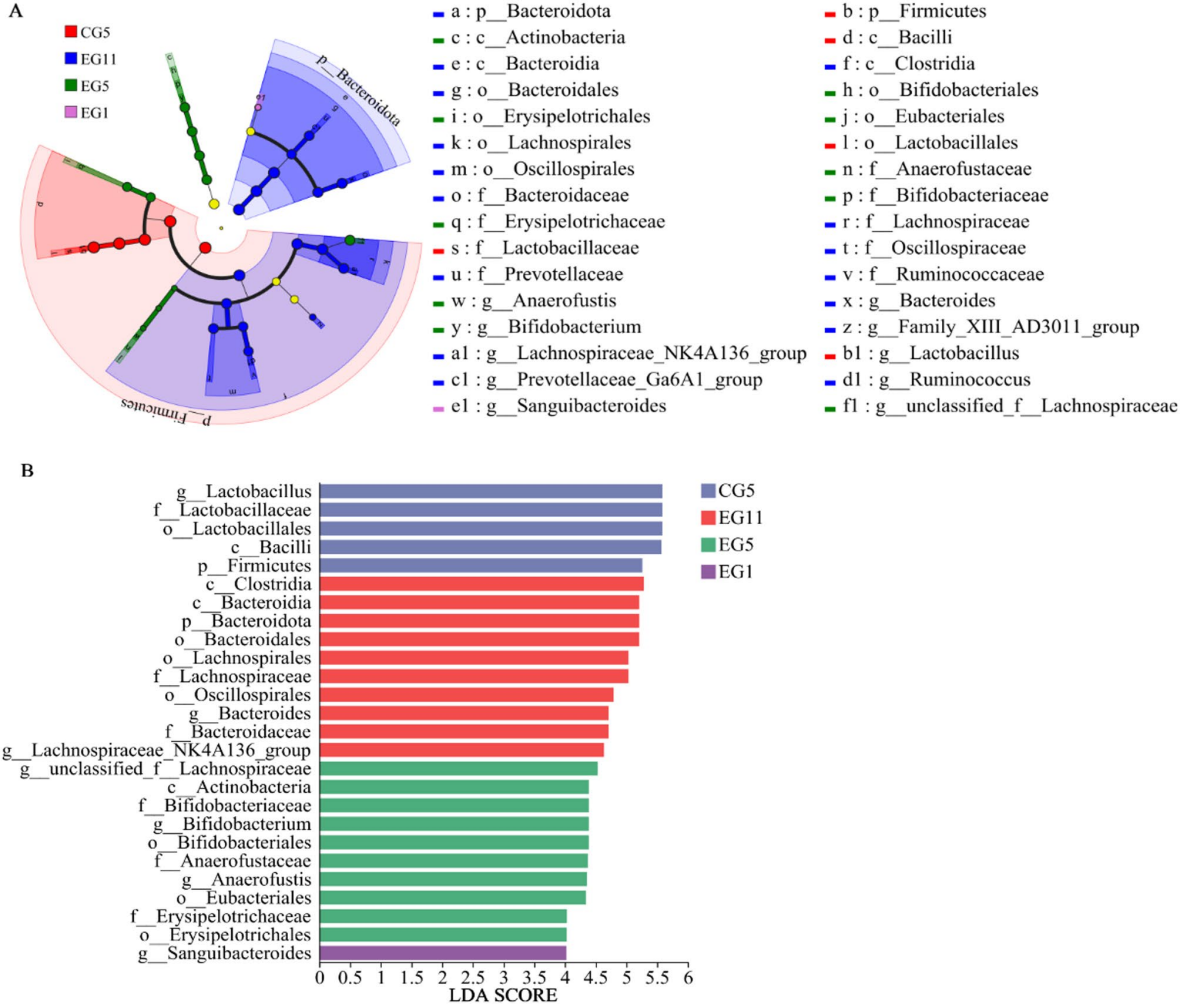
LEfSe analysis of five-toed jerboa in different treatment groups. **A** Branching diagram of the gut microbial community. **B** Linear discriminant analysis scores for each type of bacteria at the genus level, LDA > 4

To identify biomarkers that exhibited statistically significant differences between groups, we employed linear discriminant analysis (LDA) effect size (LEfSe) to screen for varying taxa at different taxonomic levels. This analysis was based on a standard LDA value greater than four. Branching diagrams were constructed from phylum to genus, revealing statistically significant differences among the CG5, EG1, EG5, and EG11 groups, each characterized by distinct bacterial taxa in the gut. A total of thirty-two biomarkers were identified through LEfSe analysis. The LDA histograms revealed that the predominant genus in the gut microflora of the CG5 group was *Lactobacillus*, whereas the EG1 group was enriched in *Sanguibacteroides*. In the EG5 group, the identified genus-level biomarkers included *Anaerofustis*, *unclassified_f_Lachnospiraceae*, and *Bifidobacterium*. For the EG11 group, the significant genera were *Bacteroides*, *Family_XII_AD3011_group*, *Lachnospiraceae_NK4A136_group*, *Prevotellaceae_Ga6A1_group*, *Ruminococcus*, and *Blautia* (Fig. 6).

### Functional prediction

**Fig. 7:**
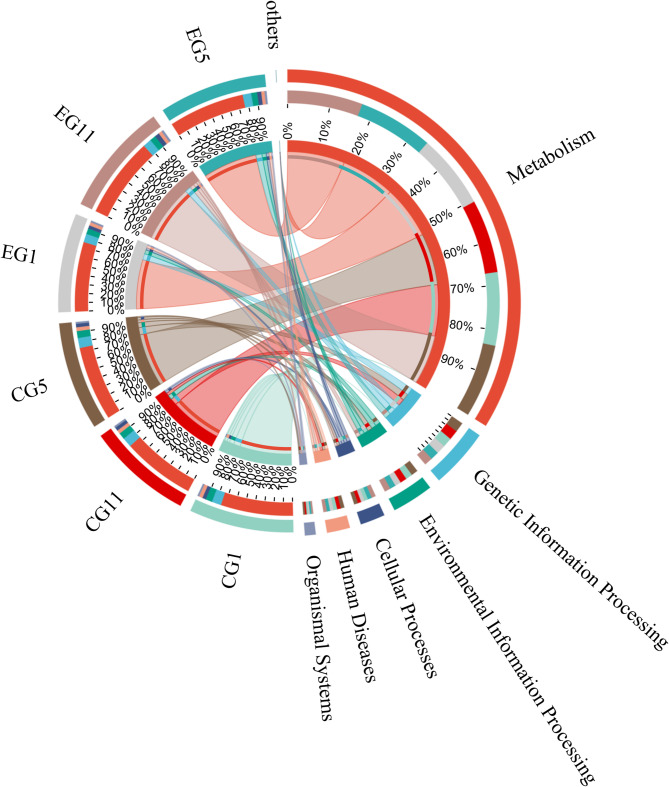
Functional prediction of the gut microbiota of the five-toed jerboa in different treatment groups. Circos plot of level 1 functional prediction

**Fig. 8:**
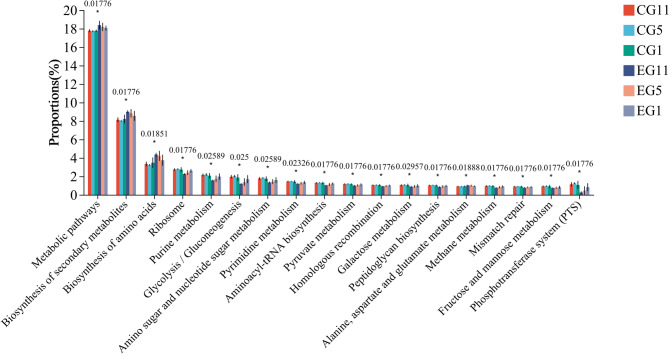
Histogram of the test for tertiary functional differences in predicted gut microbiota function in five-toed jerboa across different treatment groups

To investigate the adaptation of the five-toed jerboa to water fasting, PIRUSt2 function prediction was employed to analyze the functions of the gut microbes in all samples on the basis of 16 S rRNA data. KEGG analysis revealed that the primary functions performed by the gut microbiota were largely consistent, with the highest abundance of genes associated with metabolism, followed by genetic information processing and environmental information processing (Fig. 7). The abundance of metabolism-related genes in the CG remained relatively consistent, whereas the abundance of genes involved in metabolic and cellular processes increased in the EG as the duration of water fasting increased. More than 300 functions were predicted at level 3, and the top 21 functions with the highest abundance and a Q value < 0.05 were selected for mapping. These functions included metabolic pathways, biosynthesis of secondary metabolites, biosynthesis of amino acids, ribosomes, purine metabolism and glycolysis/gluconeogenesis. On Day 5, significant changes in functional pathways were observed in both the EG and the CG. The comparison revealed that the abundances of the pathways related to metabolic pathways; the biosynthesis of secondary metabolites; the biosynthesis of amino acids; and the metabolism of alanine, aspartic acid, and glutamate were greater in the EG than in the CG. Furthermore, the abundance of these pathways increased with increasing duration of water stress in the following order: EG11 > EG5 > EG1 (Fig. 8). This variance underscores the impact of different treatments on the functional potential of the microbiome, reflecting how water stress can influence microbial gene expression related to essential metabolic processes.

## Discussion

Many studies on wildlife have demonstrated that gut microbes can enhance an animal’s ability to adapt to environmental fluctuations [49–51]. This study utilized 16 S rRNA sequencing technology to analyze the gut microbiota of the five-toed jerboa. Our objectives were to examine the structural and functional changes in the gut microbiota of the five-toed jerboa under water stress and to investigate the role of gut microbiota in animal adaptation. Animal body weight can directly reflect an animal’s energy balance; if energy expenditure exceeds energy intake, body weight decreases, and conversely, body weight increases if energy intake surpasses expenditure. In this study, we found that water stress caused a significant decrease in the body weight of five-toed jerboa. Notably, there was a significant difference in body weight between individuals in the EG and the CG starting on the 4th day of stress exposure. The increase in energy expenditure, indicated by the decrease in body weight due to water stress, may be attributed to increased catabolism in a water-deficient environment, which promotes the breakdown of fats and proteins in the tissues. This result is supported by the findings of Feng et al., who investigated the physiological mechanisms that enable the survival of the thirteen-lined ground squirrel (*Ictidomys tridecemlineatus*), which remains chronically waterless during hibernation. They discovered that the squirrel maintains hydration by utilizing a portion of its cellular electrolytes, such as sodium, potassium, urea, and lactic acid, to regulate blood concentration during this period [52]. Additionally, Matthew et al. reported that the gut microbe-mediated urea‒nitrogen cycle during hibernation in thirteen-striped ground squirrels protects these animals, which can go without water for extended periods, by diverting urea from the kidneys and reducing the amount of water required for urine production [53]. The five-toed jerboa may also adapt to arid environments through similar gut microbial mechanisms.

In this work, we present a comparative analysis of the gut microbiota of five-toed jerboa subjected to water fasting versus those with normal water intake. Our findings indicate that the gut microbial community primarily consists of the phyla Firmicutes, Bacteroidota, and Actinobacteria, which aligns with the predominant microorganisms identified in previous studies of mammals [54]. Notably, Firmicutes and Bacteroidota were the most dominant, collectively accounting for more than 85% of the community. This observation is consistent with the results reported by Zhu et al., who reported that the intestinal microbial abundances of Firmicutes and Bacteroidota were greater in populations of *Diploderma vela* inhabiting arid regions [55]. In the present study, the gut microbiome of the EG exhibited a decrease in the abundance of Firmicutes and an increase in the Bacteroidota. This change may be closely related to the production of short-chain fatty acids (SCFAs) and their role in energy acquisition and water retention. Firmicutes are typically associated with higher SCFA production, particularly in the synthesis of butyric acid. Butyric acid not only provides energy support to the host but also plays a crucial role in regulating the osmotic pressure of the gut, thereby enhancing the host’s ability to absorb and retain water [56, 57]. However, an increase in Bacteroidota may indicate a shift in the composition of the microbial community, which could have impacted the SCFA production pattern [58]. The decrease in the ratio of Firmicutes to Bacteroidota (F/B) following water fasting treatment may be attributed to a reduction in energy absorption and the utilization efficiency of the gut microbial community in five-toed jerboas. This decrease is accompanied by an increase in the abundance of Bacteroidota, which promotes the degradation of carbohydrates and proteins, ultimately producing water during protein digestion [59]. Gut microbes in drought-adapted species, such as gerbils and camels, exhibit unique mechanisms of adaptation to arid environments. The gut microbes of gerbils efficiently break down cellulose and hemicellulose to produce SCFAs, which not only provide energy for the host but also help regulate the gut’s water retention capacity [28]. In camels, the gut microbial community shows a higher relative abundance of Firmicutes and Bacteroidota, which is similar to that of gerbils and our experimental group. Additionally, camel gut microbes can protect cellular structures and reduce water loss by producing exopolysaccharides (EPS) [60, 61]. These similarities and differences suggest that while the gut microbes of various drought-tolerant species share some functional commonalities, their specific adaptive mechanisms may differ from one species to another. Consequently, it can be inferred that changes in the abundance of these two phyla helped the five-toed jerboa to adapt to drought stress. Following the water deprivation treatment, the abundances of Actinobacteriota, Verrucomicrobia, and Proteobacteria increased in the five-toed jerboa, with individuals in the EG showing greater abundance than those in the CG. Actinobacteriota is a beneficial bacterial group known for its high metabolic potential and ability to produce a variety of biologically active molecules, making it an attractive symbiont for eukaryotic hosts [62]. This group is exposed to diverse environments and can adapt to various conditions, with metabolites isolated from arid desert environments. Multiple metabolic pathways of Actinobacteriota have been associated with adaptation to specific ecological conditions [63]. Actinobacteriota is a beneficial group of bacteria known for its high metabolic potential and ability to produce a variety of biologically active molecules, making it an attractive symbiont for eukaryotic hosts [54]. Verrucomicrobia contributes to glucose homeostasis in the human gut and possesses anti-inflammatory properties that may further support gut health. The increase in *Proteobacteria* abundance in five-toed jerboa after the water stress treatment followed the order of EG > CG. The increase in *Proteobacteria* abundance in five-toed jerboa after water deprivation treatment aligns with findings by Shin et al., who indicated that metabolic disorders leading to ecological dysregulation often result in an increase in *Proteobacteria* [64, 65]. Therefore, changes in the abundance of gut microbes such as Bacteroidota, Firmicutes, and Actinobacteriota in the gut microbiota of water-stressed individuals may facilitate their adaptation to arid environments.

The genus of gut microbes in the five-toed jerboa that was significantly influenced by water stress was *Lactobacillus* [66]. Additionally, a study by Osborne et al. on the gut microbes of African spiny mice (*Acomys cahirinus*) and golden spiny mice (*Acomys russatus*) inhabiting arid regions revealed a high abundance of *Lactobacillus* [67]. *Lactobacillus* is a crucial component of the microbiota in the gastrointestinal and oral tracts and plays a variety of physiological roles that help maintain microbial balance, improve digestion, and enhance immunity [68]. The direct interactions of *Lactobacillus* and its indirect health-promoting effects through released metabolites provide significant defense against pathogens, serving as a critical line of defense while also benefiting the host. A diet rich in prebiotics promotes the growth of *Lactobacillus*; conversely, unhealthy diets high in fat, sugar, and salt inhibit its growth [69]. In five-toed jerboa, the abundance of *Lactobacillus* in the intestine decreases under drought stress, which may weaken its protective effects on intestinal health. This reduction can impair intestinal barrier function and further affect nutrient absorption and energy metabolism. Impaired intestinal barrier function may trigger a heightened alert state in the host’s immune system, resulting in increased energy expenditure. Consequently, the EG group of five-toed jerboa exhibited a significant decrease in body weight. The decrease in Lactobacillus may impact the production of SCFAs, and a reduction in SCFAs may compel the host to derive energy from alternative sources. There was an increase in the relative abundance of genera such as *Norank_f_Muribaculaceae*, *Lachnospiraceae_NK4A136_group*, *Bacteroides*, and *Bifidobacterium*. Previous studies have identified these genera as key players in immunomodulation by beneficial bacteria. Additionally, various metabolites secreted by *Bacteroides* contribute to the stability of the immune system. These species are major producers of SCFAs in the human gut, primarily acetate and propionate, which are crucial for maintaining intestinal homeostasis [66, 70–72]. Therefore, an increase in these beneficial bacteria under drought stress could assist five-toed jerboas in adapting to drought conditions by reducing inflammation and promoting animal homeostasis. The biomarkers identified in the EG5 group included *unclassified_f__Lachnospiraceae* and *Bifidobacterium*, both of which presented relatively high relative abundances on Day 5 of water fasting. In contrast, the biomarkers found in the EG11 group included *Bacteroides*, *Lachnospiraceae_NK4A136_group*, *Prevotellaceae_Ga6A1_ group*, *Ruminococcus* and *Blautia*, which are beneficial genera whose relative abundance increased on Day 11 of water deprivation. It is possible that the five-toed jerboa mitigated dysbiosis during water fasting by promoting the growth of beneficial bacteria, increasing the abundance of SCFA-producing genera, and increasing species richness [73]. A comparison of the gut microbiomes of the red fox (*Vulpes vulpes*) and the corsac fox (*Vulpes corsac*) revealed that the corsac fox, which is better adapted to arid environments than the red fox, has higher levels of *Blautia* in its gut microbiome [74]. *Blautia* is highly correlated with drought resistance and helps the host adapt to harsh conditions. Future studies should further investigate the specific effects of these changes on host energy metabolism and water use efficiency.

In the present study, the EG demonstrated a significant increase in α diversity. This phenomenon may be closely related to the metabolic capacity of microbial communities [63, 75]. Communities with higher microbial diversity generally possess a broader metabolic potential and can utilize limited resources more efficiently [76], thereby surviving in extreme environments such as drought. Additionally, these communities typically exhibit a greater metabolic capacity to utilize scarce nutrients more effectively in resource-poor conditions [77, 78]. The α diversity of the gut microbiota changed significantly on the fifth day of water stress treatment, with greater diversity in the water stress group than in the CG. Greater microbial diversity was closely associated with greater metabolic capacity and stability in the host; a higher Shannon index indicated a more stable bacterial community and increased disease resistance. These findings suggest that the five-toed jerboa adapted to the arid environment by altering the diversity of its intestinal flora in response to water stress.

PICRUSt 2 function prediction revealed that the EG of five-toed jerboa presented an increased abundance of genes associated with metabolism and cellular processes as the duration of water stress increased. This suggests that during periods of water stress, there is a heightened demand for energy, prompting gut microorganisms to increase their metabolism and produce a greater quantity of SCFAs. In addition, the functional prediction results suggest that the upregulation of specific metabolic genes in the experimental group may align with the increased energy demands of the host. This upregulation may be linked to enhanced production of SCFAs, thereby providing additional energy support to the host. Such metabolic changes could enable the host to utilize limited resources more efficiently and sustain its viability in arid environments. This finding aligns with the research of Baniel et al., who reported that Ethiopian geladas (*Theropithecus gelada*) presented an increased proportion of bacterial genes related to energy, amino acid, and lipid metabolism during the dry season [79]. In this study, the functional potential of gut microbes was predicted using PICRUSt2. However, we recognize the limitations of this approach. PICRUSt2 relies on 16 S rRNA data and reference databases, which may not fully capture the functional diversity of microbial communities. Therefore, the results of functional predictions should be interpreted with caution. Future studies could validate these predictions through metagenomics or metabolomics techniques to more comprehensively assess the functional characteristics of microbial communities. Although significant effects were observed in this study with a limited sample size, future studies should consider increasing the sample size to enhance statistical efficacy and more comprehensively assess the role of gut microbes in five-toed jerboa regarding their drought adaptation.

In this work, water was utilized as a control variable to investigate the role of gut microorganisms in five-toed jerboa under drought stress. This study revealed that the abundance of gut microorganisms in five-toed jerboa changed in response to water stress. Furthermore, the diversity of gut microorganisms in the EG was greater than that in the CG. Additionally, the presence of beneficial bacteria in the gut microbiota of the five-toed jerboa increased under water stress, enhancing their metabolism and aiding their adaptation to drought conditions. We systematically analyzed the gut microbiome of the five-toed jerboa. This research provides a preliminary exploration of microbiome studies related to this species and offers a new perspective on how desert animals adapt to extreme arid environments through their gut microbes. The findings of this study are not only significant for basic scientific research but may also provide insights for practical applications. For instance, by identifying specific microbial functions associated with drought tolerance, we could develop microbial interventions in the future to enhance the adaptive capacity of domestic animals in arid environments. This approach could lead to new strategies for the sustainable development of livestock, particularly in water-scarce regions.

## Conclusion

In this study, we conducted experiments involving five-toed jerboa under water stress and normal drinking water intake conditions. We compared the differences in gut microbes between the two treatment groups using 16 S rRNA sequencing technology. The results indicated that the five-toed jerboa exhibited a certain level of tolerance to water stress, which significantly affected its gut microbes. Notably, the genus that was most impacted by water stress was *Lactobacillus*. The α diversity of the gut microbiome of individuals in the EG was greater than that in the CG. The gut microbes of the five-toed jerboa adapted to water-deficient environments by altering the diversity of their bacterial flora and increasing the abundance of beneficial bacteria. Changes in gut microbes that influence interactions among bacterial species are crucial for the adaptation of the five-toed jerboa to drought and water scarcity. This research provides a theoretical foundation for future studies on the mechanisms of animal adaptation to arid environments.

## Supplementary Information

Supplementary Material 1.

## Data Availability

The raw sequencing data reported in this study have been has been uploaded to the National Center for Biotechnology Information platform, with the accession number PRJNA1240187.

## Abbreviations

EG: experimental group
CG: control group
OTUs: operational taxonomic units
PCoA: principal coordinate analysis
LEfSe: linear discriminant analysis (LDA) effect size
LDA: linear discriminant analysis
