# Supplementary figures and images for: The role of gut microbes in drought adaptation in the five-toed jerboa (Orientallactaga sibirica)
Source: BMC Microbiol. 2025 Aug 5;25:482. doi: 10.1186/s12866-025-04204-z (PMC12323246; doi:10.1186/s12866-025-04204-z)

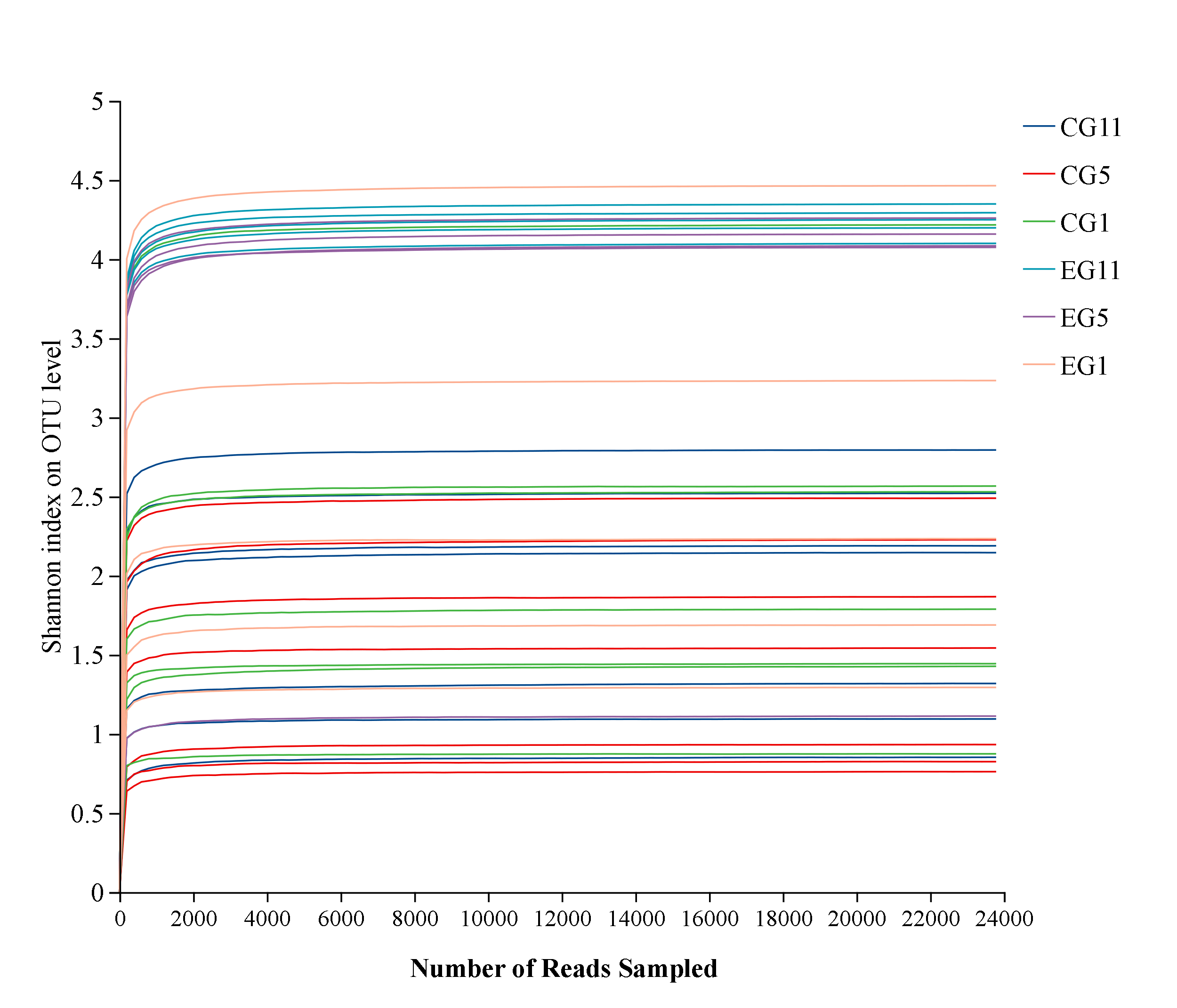


**Fig. S1** Rarefaction curves of five-toed jerboa gut microbes

Supplement: Supplementary file 1 — Supplementary Material 1. [file 12866_2025_4204_MOESM1_ESM.docx]
